# Novel *pfk13* polymorphisms in *Plasmodium falciparum* population in Ghana

**DOI:** 10.1038/s41598-022-11790-9

**Published:** 2022-05-12

**Authors:** Sena Adzoa Matrevi, Kwesi Zandoh Tandoh, Selassie Bruku, Philip Opoku-Agyeman, Tryphena Adams, Nana Aba Ennuson, Bright Asare, Oheneba Charles Kofi Hagan, Benjamin Abuaku, Kwadwo Ansah Koram, Ann Fox, Neils Ben Quashie, Andrew G. Letizia, Nancy Odurowah Duah-Quashie

**Affiliations:** 1 Department of Epidemiology, Noguchi Memorial Institute for Medical Research, College of Health Sciences, University of Ghana, Accra, Ghana; 2 West Africa Centre for Cell Biology and Infectious Pathogens, Department of Biochemistry Cell and Molecular Biology, College of Basic and Applied Sciences, University of Ghana, Accra, Ghana; 3 United States Naval Medical Research Unit 3, Ghana Laboratory, Accra, Ghana; 4 Centre for Tropical Clinical Pharmacology and Therapeutics, University of Ghana Medical School, College of Health Sciences, University of Ghana, Accra, Ghana

**Keywords:** Computational biology and bioinformatics, Genetics, Molecular biology, Medical research

## Abstract

The molecular determinants of *Plasmodium falciparum* artemisinin resistance are the single nucleotide polymorphisms in the parasite’s kelch propeller domain, *pfk13*. Validated and candidate markers are under surveillance in malaria endemic countries using artemisinin-based combination therapy. However, *pfk13* mutations which may confer parasite artemisinin resistance in Africa remains elusive. It has therefore become imperative to report all observed *pfk13* gene polymorphisms in malaria therapeutic efficacy studies for functional characterization. We herein report all novel *pfk13* mutations observed only in the Ghanaian parasite population. In all, 977 archived samples from children aged 12 years and below with uncomplicated malaria from 2007 to 2017 were used. PCR/Sanger sequencing analysis revealed 78% (763/977) of the samples analyzed were wild type (WT) for *pfk13* gene. Of the 214 (22%) mutants, 78 were novel mutations observed only in Ghana. The novel SNPs include R404G, P413H, N458D/H/I, C473W/S, R529I, M579T/Y, C580R/V, D584L, N585H/I, Q661G/L. Some of the mutations were sites and ecological zones specific. There was low nucleotide diversity and purifying selection at the *pfk13* locus in Ghanaian parasite population. With increasing drug pressure and its consequent parasite resistance, documenting these mutations as baseline data is crucial for future molecular surveillance of *P. falciparum* resistance to artemisinin in Ghana.

## Introduction

Malaria remains a challenge in Africa, where about 94% of global malaria morbidity and mortality occur^1^. The most virulent malaria parasite, *Plasmodium falciparum* is resistant to most antimalarials. In order to slow the development of resistance in the parasite, the use of artemisinin-based combination therapy (ACTs) was introduced in malaria endemic countries by the World Health Organization (WHO)^2^. However, the report of decreased artemisinin (ART) efficacy in Southeast Asia (SEA)^3,4^ is a huge impediment to disease control efforts. The discovery of mutations in the *P. falciparum* kelch propeller domain on chromosome 13 (*pfk13*) as markers has helped tremendously in molecular surveillance in malaria endemic countries^4^. The *pfk13* validated markers include F446I, N458Y, M476I, Y493H, R539T, I543T, P553L, R561H, P574L and C580Y^1,5,6^. Other markers yet to be validated are P441L, G449A, C469F/Y, A481V, R515K, P527H, N537I/D, G538V, V568G, R622I and A675V^1^. The list of validated *pfk13* resistant SNPs is increasing and updates are done by the WHO over time.

In Africa, molecular surveillance studies have reported several SNPs, including M472I, Y558C, K563R, P570L, P615S in Niger^7^, R622I in Ethiopia^8^, C473F in Senegal^9^, F434S, F442F, I684N in Nigeria^10^ and M472I, A569T in the Democratic Republic of Congo^11^. These observed SNPs, although they have not yet been functionally characterized to determine their role in ART resistance. However, these have to be documented because of the possibility that they could be selected with increasing drug pressure and become the markers of ART resistance in Africa. This scenario is possible because of the reported local emergence of *pfk13* mutations in the Amazonia^12,13^.

*Pfk13* SNPs reported from other disease endemic countries including some of the validated SNPs and their variants which were observed in Ghanaian malaria parasites from the same samples have already been published^14^. In this report we document all the novel SNPs that have been observed only in the Ghanaian parasite population because these could be of interest in the future. These SNPs have not been reported from any country as revealed from searches in published articles from PUBMED up to the date of submitting this article.

## Results

Twenty-two percent of the total number of samples (214/977) had *pfk13* mutations, of which 78 were unique SNPs and 95% of those were non-synonymous. Mutations were observed in 63 codons and ranged from one SNP per codon to three SNPs per codon (N458D, N458I, N458H). Most of the novel SNPs were seen in only one sample (frequency of 0.47%). The coastal zone consisting of Accra and Cape-Coast (which are also urban areas) had more novel SNPs than the forest (having 6 sites—Begoro, Bekwai, Koforidua, Hohoe, Tarkwa, Sunyani). Of the sites in the forest zone, Koforidua had the most novel SNPs compared to other sites of the same zone. All the novel SNPs are shown in Table 1. Unique mutations were observed at the different sites and ecological zones. The ecological zone unique SNPs are, C580R and K669E/N for coastal, M579T/Y and D584L for forest and N554P and A569P for the savannah.

**Table 1:** Novel non-synonymous SNPs at the sentinel sites. All novel mutations have been cited under the domains of the kelch propeller region showing the codons with the mutation, the base changes and the sentinel site where they were observed.

Domains	Codons	Reference amino acid	Observed amino acid	Mutation	Specific base change	Site
**BTB/POZ**	404	R	G	AGA → GGA	A → G	Wa
	411	R	K	AGA → AAA	G → A	Hohoe
	412	N	I	AAT → ATC	AT → TC	Navrongo
	412	N	S	AAT → AGT	A → G	Begoro
	413	P	H	CCG → CAC	CG → AC	Hohoe
	422	L	F	TTC → CTC	T → C	Wa
	431	E	Q	GAA → CAA	G → C	Hohoe
	432	A	T	GCA → ACA	G → A	Hohoe
	442	F	L	TTC → CTC	T → C	Hohoe
**Blade 1**	445	V	L	GTA → CTA	G → C	Begoro, Wa
	455	E	K	GAA → AAA	G → A	Hohoe, Koforidua, Wa
	456	Y	N	TAT → AAT	T → A	Accra
	458	N	D	AAT → GAT	A → G	Koforidua
	458	N	I	AAT → ATT	A → T	Koforidua
	458	N	H	AAT → CAT	A → C	Koforidua
	461	E	G	GAA → GGA	A → G	Accra
	469	C	R	TGC → CGC	T → C	Cape Coast
	470	W	R	TGG → CGG	T → C	Cape Coast
	473	C	W	TGT → TGG	T → G	Cape Coast
	473	C	S	TGT → TCT	G → C	Hohoe
**Blade 2**	485	S	G	AGT → GGT	A → G	Koforidua
	488	L	F	TTG → TTC	G → C	Cape Coast, Hohoe
	494	V	I	GTT → ATT	G → A	Hohoe
	499	N	T	AAC → ACC	A → C	Hohoe
	510	V	G	GTG → GGG	T → G	Navrongo
	510	V	L	GTG → TTG	G → T	Wa
	522	S	R	AGT → AGG	T → G	Cape Coast
**Blade 3**	523	N	K	AAT → AAA	T → A	Sunyani
	529	R	K	AGA → AAA	G → A	Cape Coast
	529	R	I	AGA → ATA	G → T	Koforidua
	530	N	I	AAT → ATT	A → T	Sunyani
	530	N	S	AAT → TCA	AAT → TCA	Koforidua
	532	C	S	TGT → AGT	T → A	Begoro
	536	S	A	TCA → GCA	T → G	Bekwai
	547	D	E	GAT → GAA	T → A	Cape Coast
	547	D	N	GAT → AAT	G → A	Hohoe
	554	N	P	AAT → CCT	AA → CC	Accra
	554	N	T	AAT → ACT	A → C	Hohoe
	555	V	A	GTA → GCA	T → C	Sunyani
	556	E	D	GAA → GAT	A → T	Sunyani
	559	D	Y	GAT → TAT	G → T	Navrongo
	565	W	R	TGG → CGC	TG → CC	Begoro
	569	A	P	GCA → CCA	G → C	Wa
**Blade 4**	578	A	P	GCT → CCT	G → C	Wa
	579	M	T	ATG → ACG	T → C	Koforidua
	579	M	Y	ATG → TAT	ATG → TAT	Cape Coast
	580	C	V	TGT → GTG	TGT → GTG	Cape Coast
	580	C	R	TGT → CGT	T → C	Begoro
	584	D	L	GAT → TTG	GAT → TTG	Koforidua
	585	N	H	AAT → CAT	A → C	Hohoe
	585	N	I	AAT → ATA	AT → TA	Koforidua
	587	I	T	ATT → ACT	T → C	Cape Coast
	587	I	N	ATT → AAT	T → A	Cape Coast, Koforidua
	590	I	T	ATT → ACT	T → C	Navrongo
	598	L	I	TTA → ATA	T → A	Bekwai
	605	E	D	GAA → GAC	A → C	Cape Coast
	615	P	L	CCA → CTA	C → T	Cape Coast
**Blade 5**	616	Y	H	TAT → CAT	T → C	Koforidua
	619	L	V	TTA → GTA	T → G	Begoro
	623	S	N	AGT → AAT	G → A	Cape Coast
	628	F	L	TTT → CTT	T → C	Cape Coast
	628	F	L	TTT → TTA	T → A	Hohoe, Koforidua
	633	Q	H	CAA → CAT	A → T	Accra
	640	I	F	ATT → TTT	A → T	Cape Coast
	640	I	S	ATT → AGT	T → G	Begoro
	643	E	D	GAA → GAC	A → C	Cape Coast
	646	I	K	ATA → AAA	T → A	Cape Coast
	648	D	Y	GAT → TAT	G → T	Sunyani
	661	Q	L	CAA → CTA	A → T	Cape Coast, Koforidua
	661	Q	G	CAA → GCA	CA → GC	Koforidua
	664	N	H	AAT → CAT	A → C	Cape Coast
**Blade 6**	668	E	D	GAG → GAT	G → T	Cape Coast, Koforidua
	669	K	E	AAA → GAA	A → G	Cape Coast, Koforidua
	669	K	N	AAA → AAC	A → C	Koforidua
	672	N	I	AAT → ATT	A → T	Cape Coast, Koforidua
	690	G	D	GGC → GAC	G → A	Cape Coast, Koforidua
	692	V	L	GTT → CTT	G → C	Cape Coast
	696	C	S	TGT → AGT	T → A	Cape Coast

### Distribution of mutations in the *pfk13* propeller domain in the Ghanaian isolates

Novel SNPs which were unique to the various sites were observed at different domains of the propeller region. The SNPs exclusive to Hohoe were mostly located within the BTB/POZ domain to blade 3 and those of Koforidua were located within blades 3 to 6. SNPs observed in the samples from Cape Coast were located within blades 4, 5 and 6 and those for Accra were found in blades 1, 3 and 5. Of the 78 novel mutations detected, the highest number of mutations were recorded in blade 3 and the least number in blades 2 and 6 as shown in Table 1.

### *P. falciparum* k13 gene showed low diversity and evidence of purifying selection in Ghanaian parasite population

**Figure 1:**
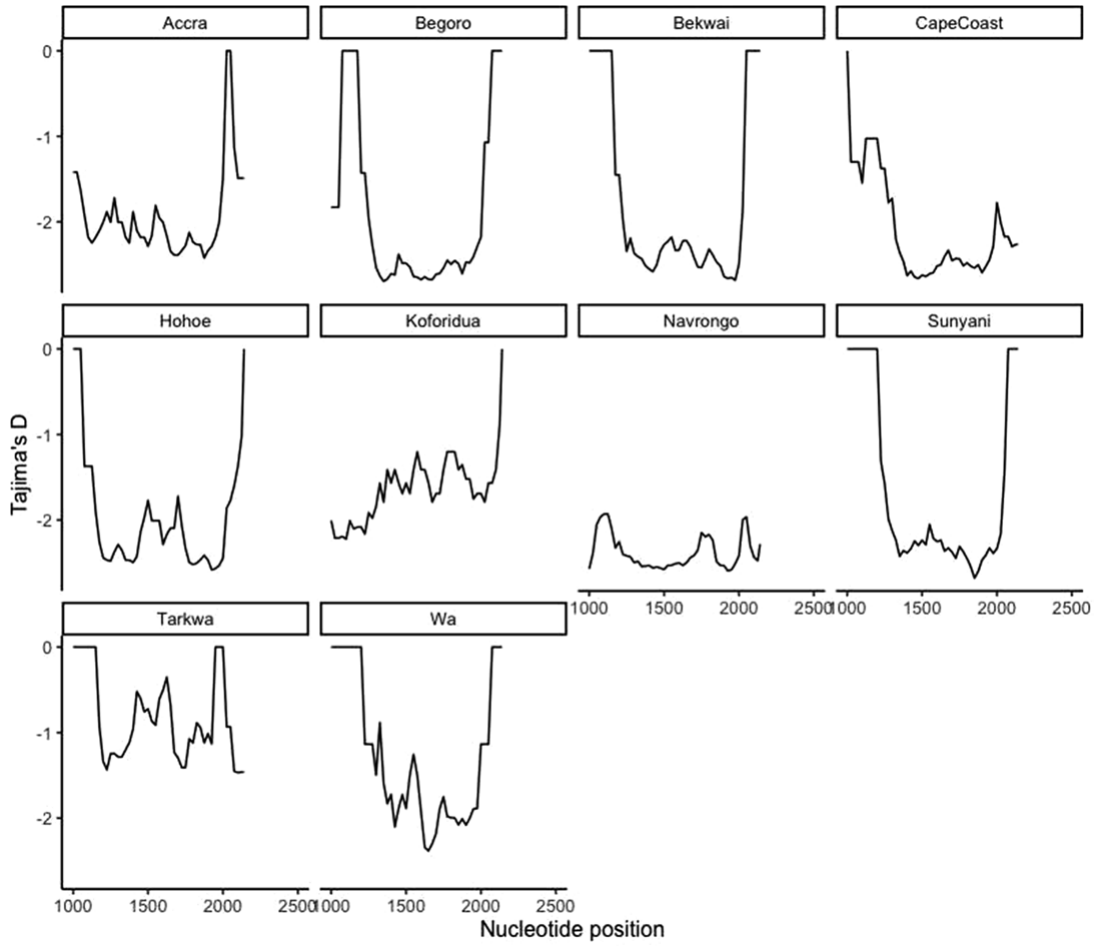
Sliding window plot of Tajima’s D for the pfk13 gene showing distribution by location/site. The computational analysis of the sequences to reveal the nucleotide diversity of the mutations in the pfk13 gene in Ghanaian isolates by study sites. Nucleotide positions is from 1000 to 2181 bp. Window length is 100 bp and step size is 25 bp.

**Figure 2:**
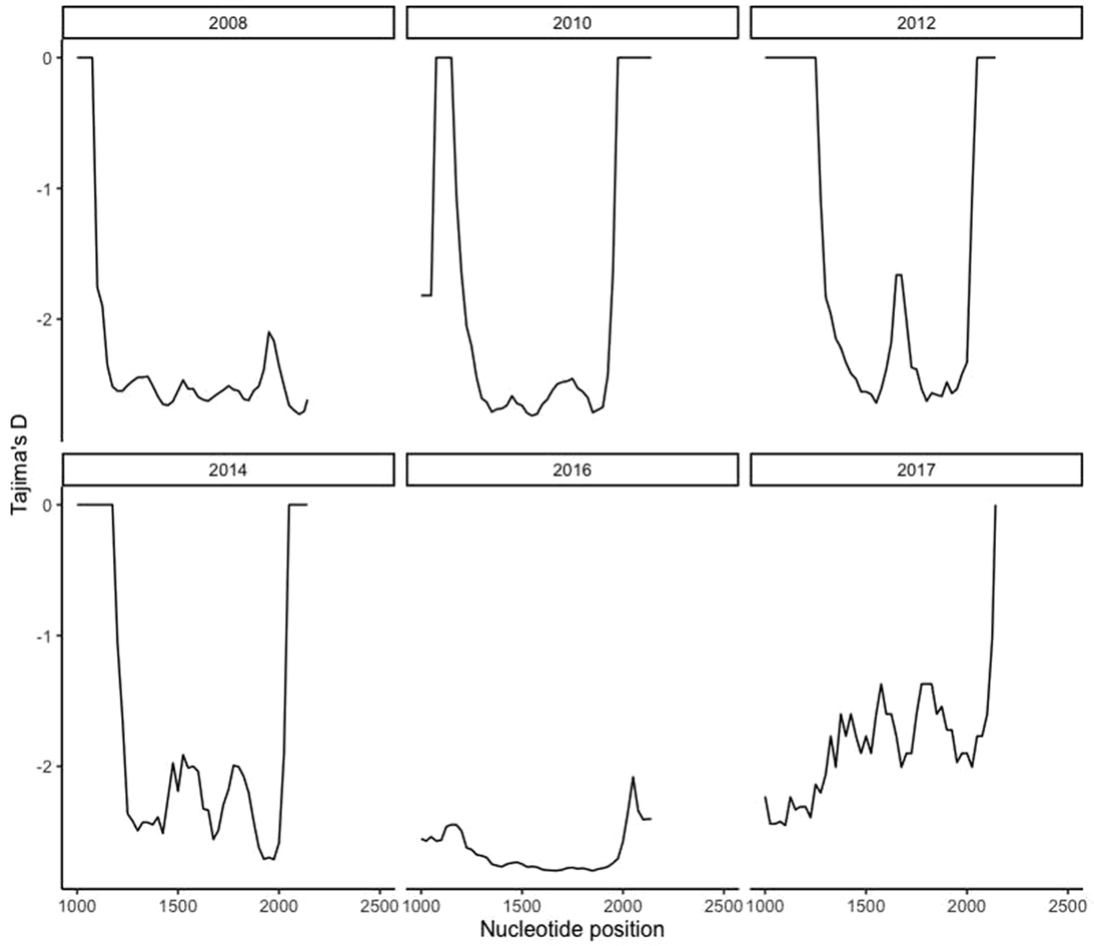
Sliding window plot of Tajima’s D for the pfk13 gene showing temporal distribution. The computational analysis of the sequences to reveal the nucleotide diversity of the mutations in the pfk13 gene in Ghanaian isolates by year.Nucleotide positions is from 1000 to 2181 bp. Window length is 100 bp and step size is 25 bp.

**Figure 3:**
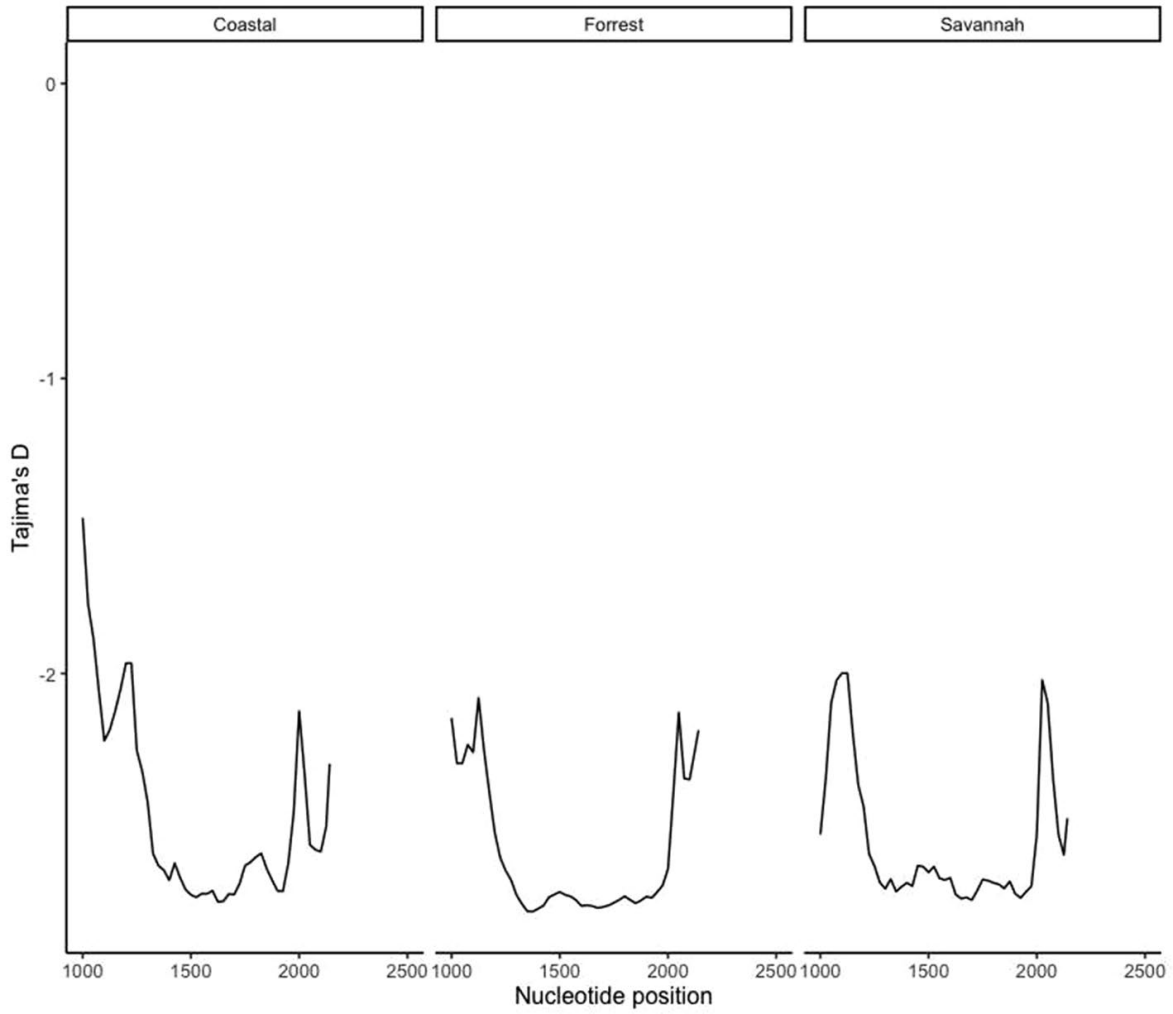
Sliding window plot of Tajima’s D for the pfk13 gene showing distribution by ecological zones. The computational analysis of the sequences to reveal the nucleotide diversity of the mutations in the *pfk13* gene in Ghanaian isolates by ecological zones. Nucleotide positions is from 1000 to 2181 bp. Window length is 100 bp and step size is 25 bp.

To investigate the diversity at the *pfk13* locus, we determined population genetics metrics of DNA polymorphism using the 792 sequences in total. Overall genetic diversity at the *pfk13* locus was low (π = 0.00383) (Table 2) and indicates that the gene locus sequence among the 792 samples analyzed was largely similar. This similarity or low genetic diversity did not change when analyzed per location, year, or ecological zone (Table 2; Figs. 1, 2, 3). Additionally, polymorphism measured by the number of segregating sites was 1034. The investigation of the evidence of selection acting on the *pfk13* locus using the site frequency spectrum metric, Tajima's D was done. Positive values of Tajima's D are suggestive of balancing selection and negative values of purifying or negative selection. The analysis shows that the *pfk13* gene, for the period and locations analyzed, was under purifying selection. A total of 307 haplotypes were found in the gene locus with a haplotype diversity of 0.6887. The forest ecological zone contributed the highest number of haplotypes (h = 159) and the year 2016 reported the highest number of haplotypes (h = 186).

**Table 2:** Summary of computational analysis of DNA polymorphisms found in *pfk13* in Ghanaian isolates by location, year and ecological zones. The computational analysis of the sequences to reveal the nucleotide diversity of the mutations in the *pfk13* gene in Ghanaian isolates for study sites, year and ecological zones.

	π	S	Tajima's D	T’s D *p* value	No. of haplotypes/haplotype diversity (h/Hd)
**A. Location**					
Accra	0.00395	136	− 2.70944	< 0.001	29 (0.926)
Begoro	0.00418	222	− 2.80559	< 0.001	30 (0.759)
Bekwai	0.00589	232	− 2.66217	< 0.001	28 (0.754)
CapeCoast	0.00315	247	− 2.87368	< 0.001	48 (0.722)
Hohoe	0.00154	138	− 2.89841	< 0.001	44 (0.686)
Koforidua	0.00096	102	− 2.70784	< 0.001	28 (0.472)
Navrongo	0.00299	276	− 2.91636	< 0.001	41 (0.607)
Sunyani	0.00415	164	− 2.64914	< 0.001	22 (0.614)
Tarkwa	0.01467	89	− 1.15583	> 0.10	6 (1.000)
Wa	0.00303	87	− 2.55226	< 0.001	24 (0.906)
Total	0.00383	1034	− 2.85874	< 0.001	307 (0.6887)
**B. Year**					
2007	0.00919	305	− 2.78904	< 0.001	23 (0.829)
2010	0.00798	344	− 2.74562	< 0.001	32 (0.666)
2012	0.00255	132	− 2.80328	< 0.001	18 (0.468)
2014	0.00688	255	− 2.57399	< 0.001	51 (0.879)
2016	0.00289	767	− 2.9292	< 0.001	186 (0.7127)
2017	0.00097	102	− 2.92075	< 0.001	28 (0.474)
Total	0.00383	1034	− 2.85874	< 0.001	307 (0.6887)
**C. Ecological zones**					
Coastal	0.00504	460	− 2.9099	< 0.001	82 (0.827)
Forest	0.00326	706	− 2.88742	< 0.001	159 (0.6300)
Savannah	0.00458	491	− 2.94234	< 0.001	71 (0.731)
Total	0.00383	1034	− 2.85874	< 0.001	307 (0.6887)

**S**—number of segregating sites in the gene; **π—**nucleotide diversity at the gene locus.

## Discussion

The need to report all observed SNPs in the *pfk13* gene is important especially when the molecular markers for resistance in Africa are yet to be revealed. From this study, sequence analysis revealed a number of novel SNPs observed only in the Ghanaian parasite population over a decade. Although the mutations are many in different codons of the gene locus, the frequencies were low and the computational DNA analysis showed low nucleotide diversity in the population which is under purifying selection. Our previous paper has already reported mutations seen in Ghanaian isolates that have been observed elsewhere including variants of some of the validated and candidate markers of ART resistance^14^. Functional characterisation using CRISPR Genome Editing Technology followed by Ring Stage Survival Assay (RSA) of two clones with one novel mutation, C580R; C580R_1 and C580R_2 showed parasite survival rates of 18% and 14% respectively and that of the validated marker, C580Y, was 28% in the same experiment (OCK Hagan et al., data yet to be published). The findings of this experiment support the fact that all observed mutations in *pfk13* could be potential markers of drug resistance and therefore must be documented.

The unique mutations observed in the parasite population of Ghana were not shared, even among sites of the same ecological zone and could be a reflection of minimum gene flow between the sites within each zone^12^. This observation corroborates the findings from data available on resistance to ART. The data do not show a cluster of mutations geographically and there is lack of sharing of common mutations among parasite populations thereby resulting in regional diversity^15–17^. The novel mutations are as a result of genetic recombination and localised evolution of the gene, which is a consequence of high transmission intensity. The differences in the transmission patterns^18–20^ could be a probable explanation to the observed genetic variability. Inadvertently, most of the SNPs were observed in one sample and only a few were seen in 2 or 3 samples. The fact that they were non-synonymous mutations could also be affecting the fitness cost of the parasites and may not necessarily be linked to drug resistance. In addition, it could be an evidence of the start of an independent emergence of *pfk13* mutations in Ghana as observed in the parasite population of Guyana^12,16^.

The mutations in the Ghanaian isolates were distributed in all the domains, from the BTB/POZ to blade 6 with variations in sentinel sites located in the same ecological zone. Most mutations were in blade 3 followed by blades 4 and 5 but with low frequencies. The propeller domain is known to be conserved in *P. falciparum,* however, the mutations observed could be parasite adaptation due to selective pressures of antimalarial drugs use in Africa (fake drugs, noncompliance and presumptive treatment of malaria)^13,21^. The large pool of low frequency genetic mutations could help with the emergence of resistance faster than anticipated due to increasing drug pressure from ACT use^22^. Unlike the high frequency of non-synonymous mutations in parasites of the SEA region moving from intermediate to fixation levels, those of Africa occur at very low frequencies with high allelic variation^23^.

Nucleotide diversity (π) at the *pfk13* locus can be considered an indirect measure of the potential for the selection of an ART tolerant variant. A high π at the *pfk13* suggests sufficient diversity for a soft or hard selection sweep on the locus. In contrast, a low π suggests a reduced probability for a selection sweep on the *pfk13* locus. The finding of low diversity at the *pfk13* locus in this regard suggests that the risk of a tolerant *pfk13* variant emerging between 2007 and 2017 was low. It is also evident that *pfk13* is largely conserved in the *P. falciparum* population of Ghana. This lack of diversity at the *pfk13* locus may be due to the fitness cost of any new variant.

Within the context of relatively high transmissions that correlate with higher sexual outcrossing in the mosquito vector and thus the breakdown by recombination of any nascent *pfk13* variant/haplotype, our findings are expected. Other factors that might mitigate against high diversity in the *pfk13* locus include the prevalence of human malaria immunity and within-host multiplicity of infection/competition. These factors may act to negatively select emerging ART tolerant variants segregating in our population as portrayed by the results. The finding of negative Tajima’s D may also suggest recent population expansion with multiple low-frequency variants. This presence of several variants at low frequencies contributes to the haplotype diversity observed in the analysis. Additionally, the findings of low nucleotide diversity and purifying selection at the *pfk13* locus is congruent with the findings of a similar study that investigated the evolution and genetic diversity of the *pfk13* gene^24^.

## Conclusion

A change in genetic composition and the resultant change in amino acids affects protein function. The observation of numerous novel mutations which are non-synonymous with low frequencies is indicative of the development of a nascent resistance at the genotypic level yet to be revealed as phenotypic traits in Ghanaian parasites. The current reported efficacies of ACTs is above 95%^25^ which is quite high as compared to some countries in the region. The novel mutations would be monitored continuously and functional characterization would be performed on those with increasing frequencies over time to establish their role in parasite resistance to ACTs in Ghana.

## Methods

### Study sites and population

**Figure 4:**
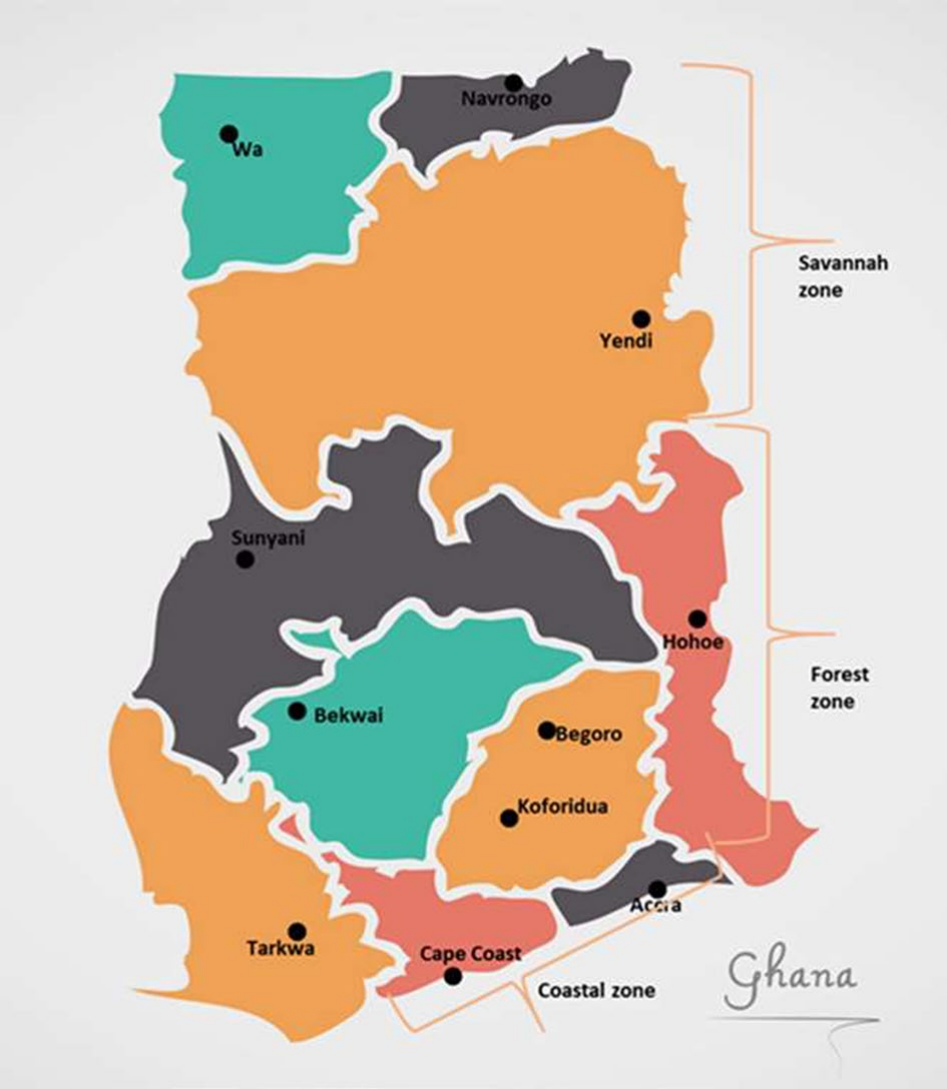
A map of Ghana showing the study sites in the ecological zones. These sites are designated for antimalarial drug therapeutic efficacy studies in all regions of Ghana.

Archived samples from therapeutic efficacy studies (TES) conducted in sentinel sites in three different ecological zones of Ghana namely coastal, forest and savannah were used for the study. Perennial transmission of malaria occurs in the coastal and forest zones and seasonal malaria transmission occurs in the savannah zone. The sentinel sites are Accra, Begoro, Bekwai, Cape-Coast, Hohoe, Koforidua, Navrongo, Sunyani, Tarkwa, Yendi and Wa (Fig. 4). Accra and Cape-Coast lie in the coastal savannah zone; Navrongo, Yendi and Wa lie in the guinea savannah zone; Begoro, Bekwai, Koforidua, Sunyani, Hohoe and Tarkwa lie in the forest zone. The information on the study sites is well documented in Matrevi et al.^14^.

### Samples and molecular analysis

Archived filter paper blood blots, prepared from children 12 years and below reporting at the clinic with uncomplicated malaria from 2007 to 2017 malaria transmission season were used. The parents/guardians of the children gave informed consent for their participation in the studies. The consent also covered the future use of the archived samples for further molecular analysis. DNA was extracted using a QIAamp DNA Mini Kit (QIAGEN, Germany) following the manufacturer’s protocol. Targeted portion of *pfk13* gene was amplified using the nested PCR protocol by Talundzic et al.^26^ with minor modifications. Positively amplified samples were Sanger sequenced by Macrogen, Europe (Netherlands).

### Sequence analysis

Obtained sequences from the *pfk13* genes were submitted to the standard nucleotide basic local alignment search tool (BLAST) database search program of the National Center for Biotechnology Information (NCBI) website to determine the authenticity of the sequences. The sequences were then aligned using 3D7 wild type *pfk13* sequence (PF3D7_1343700) for reference obtained from PlasmoDB (www.Plasmodb.org). Sequences were edited using BioEdit ClustalW Multiple Sequence Alignment Software. They were further analysed using CLC Main Workbench 20.04 software (Qiagen, Aarhus, Denmark) and Benchling.com (San Francisco, CA, USA). Other single nucleotide polymorphisms were searched for using PubMed tool for new SNPs published by other researchers.

### Computational pipeline for population genetics analysis of *pfk13* gene

Base-calling, alignment, and deconvolution of Sanger chromatogram trace files were done using the command-line version of the application Tracy^27^. The output binary variant call format (bcf) files for each sample were converted to human-readable variant call format (vcf) files using custom bash scripts. Low-quality variants (< 40) and indels were filtered out from the vcf file. After this, vcf files were merged and variants extracted and annotated into a text file using custom bash scripts, SnpEff (v4.1), and vcftools. Fasta files were generated using custom bash scripts and fed into DnaSP6.0 to determine the DNA polymorphisms metrics and Tajima's D^28^.

### Ethics declarations

The study protocol was approved by the Institutional Review Boards of the Noguchi Memorial Institute for Medical Research (NMIMR) and Naval Medical Research Center in compliance with all applicable federal regulations governing the protection of human subjects of the US Government. The IRB protocol number is 032/05-06a amed. 2021.

## Data Availability

All data generated or analyzed during this study are included in this published article. Raw sequence data is available upon reasonable request from the corresponding author.
